# Tailoring the Geometry of Bottom-Up Nanowires: Application to High Efficiency Single Photon Sources

**DOI:** 10.3390/nano11051201

**Published:** 2021-05-01

**Authors:** Dan Dalacu, Philip J. Poole, Robin L. Williams

**Affiliations:** National Research Council Canada, Ottawa, ON K1A 0R6, Canada; philip.poole@nrc.ca (P.J.P.); robin.williams@nrc.ca (R.L.W.)

**Keywords:** nanowires, quantum dots, single photon sources

## Abstract

For nanowire-based sources of non-classical light, the rate at which photons are generated and the ability to efficiently collect them are determined by the nanowire geometry. Using selective-area vapour-liquid-solid epitaxy, we show how it is possible to control the nanowire geometry and tailor it to optimise device performance. High efficiency single photon generation with negligible multi-photon emission is demonstrated using a quantum dot embedded in a nanowire having a geometry tailored to optimise both collection efficiency and emission rate.

## 1. Introduction

High efficiency sources of single photons and entangled photon pairs are a required resource in many quantum information processing applications [1]. Sources based on semiconductor quantum dots offer deterministic operation as well as high collection efficiencies through appropriately designed photonic nanostructures [2,3]. Devices based on quantum dots in bottom-up nanowires are particularly attractive. They are readily grown using site-selective techniques [4] with each nanowire containing only one emitter [5] naturally positioned for optimal coupling to the optical mode supported by the nanowire [6].

**Figure 1:**
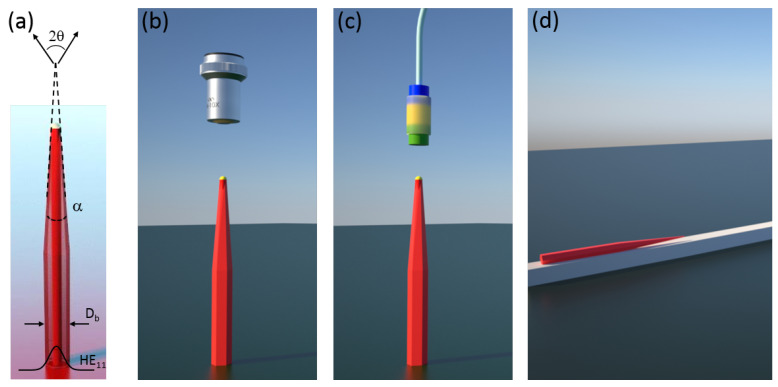
(**a**) Schematic of a nanowire-based quantum dot single photon source. Different implementations of the source: (**b**) Free-space coupled. (**c**) Fibre-coupled using graded index lenses. (**d**) On-chip using evanescent coupling of the nanowire optical mode to an underlying ridge waveguide.

In order to achieve the highest emission rates and collection efficiencies in nanowire sources [7] careful consideration of the nanowire geometry, shown in Figure 1a, is required [8]. Ideally, the nanowire base diameter $D_{b}$ is chosen such that only the fundamental waveguide mode ${\mathrm{HE}}_{11}$ is confined and the spontaneous emission rate of the emitter into this mode is maximised. Efficient collection of the mode using limited numerical aperture (NA) optics requires its gradual expansion to reduce the far-field emission angle. Tapered nanowires with taper angles α< 2° can provide near unity collection of the emitted mode using available optics and suppresses reflection from the top of the nanowire [9].

Figure 1b–d shows three device configurations with different taper requirements for high collection efficiency operation. Collection using free-space optics (Figure 1b) places the least stringent requirements on the source as large NA objectives are readily available. An all-fibre approach, however, requires less divergent sources due to the limited availability of high NA optical fibres. The requirements can be relaxed using appropriate sets of graded index lenses [10] as depicted in Figure 1c. For on-chip sources shown in Figure 1d, an appropriately designed taper will facilitate mode transfer from the nanowire to an underlying ridge waveguide [11].

In this work we describe an approach for controlling the geometry in bottom-up nanowires based on combining vapour-liquid-solid (VLS) and selective-area (SA) epitaxy. We first review the SA-VLS growth technique and then demonstrate how the technique can be used to facilitate the growth of nanowires having geometries with specified base diameters and tailored tapers. The impact of taper geometry on collection efficiency is demonstrated using a quantum dot emitter embedded in the nanowire. Finally, we demonstrate high purity single photon generation from a quantum dot embedded in a nanowire with an optimised geometry.

## 2. Methods

**Figure 2:**
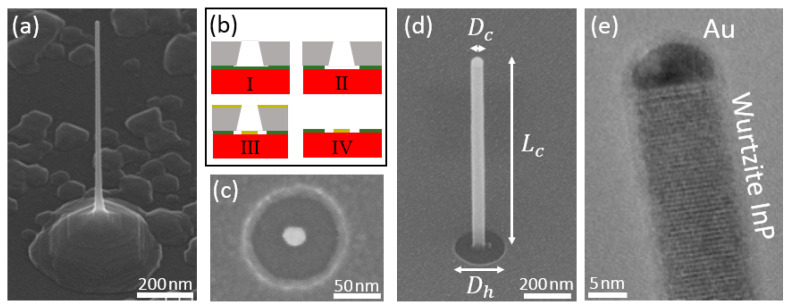
(**a**) Scanning electron microscopy (SEM) image viewed at 45° of a VLS nanowire grown without a selective oxide mask. (**b**) Process for fabricating the patterned substrate used in SA-VLS epitaxy showing the semiconductor substrate (red), the oxide mask (green), the electron-beam resist (grey) and the metal catalyst (yellow). (**c**) Plan-view SEM image of the patterned substrate. (**d**) SEM and (**e**) transmission electron microscopy image of a template nanowire.

In SA-VLS epitaxy of nanowires [12] the growth substrate is prepared with metal catalysts centred in the middle of openings in a dielectric mask on a semiconductor substrate. The process for fabricating the patterned substrate is shown in Figure 2b. A 20 nm thick film of SiO${}_{2}$ is deposited on a (111)B InP substrate using plasma-enhanced chemical vapour deposition. This is coated with electron-beam resist and patterned to expose a circular hole. The oxide is then wet-etched through the hole in the resist using hydrofluoric acid to expose the InP surface with the oxide hole size determined by the etch time. Finally, 5 nm of gold is deposited in the centre of the hole using a self-aligned lift-off process. In Figure 2c, a scanning electron microscopy (SEM) image of the resulting structure shows a 20 nm gold catalyst in the centre of the oxide opening surrounded by an annulus of exposed InP substrate.

Growth on such a patterned substrate is demonstrated for InP nanowires using chemical beam epitaxy with trimethylindium (TMI) and pre-cracked PH${}_{3}$ sources. In a first growth step, we use conditions [13] that promote VLS epitaxial growth (i.e., material incorporation occurs primarily at the gold-semiconductor interface) with minimal incorporation on the substrate annulus surrounding the gold. Specifically, we use low growth temperatures, typically 420 °C to 435 °C, and a low Group V flux. For relatively short growth times, these growth conditions produce untapered nanowires with diameters determined by the the size of the gold catalyst, $D_{c}$, as shown in Figure 2d. Additionally, the growth rate, controlled by the TMI flux, is kept low, which was shown to promote the growth of pure wurtzite phase nanowires with a minimal number of stacking faults [14], see Figure 2e.

We refer to the structures produced in this first growth step as the nanowire template as it serves as a template for subsequent growth steps which define the taper geometry, discussed in Section 3. Using such a SA-VLS nanowire template has several advantages:

(i) Without the selectivity provided by the oxide mask, parasitic growth on the substrate surface, typically more pronounced within a Group III migration length from the nanowire, leads to a pedestal structure shown in Figure 2a. The complex competition between pedestal and nanowire material incorporation makes it difficult to tailor the geometry of the nanowire. With the oxide mask, the regions where material can incorporate are defined through lithography, allowing not only for calibrating the amount of material required for a certain structure, but also to define that structure through control of the size of the opening.
(ii) Since shell growth occurs epitaxially, the crystal phase of the final structure is determined by the phase of the template nanowire. One can therefore grow nanowires with different diameters by varying the shell thickness without affecting the crystal phase. This is in contrast to growth solely using VLS, where the crystal phase has been shown to depend on the catalyst diameter [6,15].
(iii) The separation of the growth process into a template step and a shell growth step facilitates designing different sections of the nanowire that have distinct geometric requirements. For the case of single photon sources, quantum dots incorporated in the template nanowire are required to be sufficiently small to provide strong confinement of the electron and hole wavefunctions. The clad nanowire (also referred to as the photonic nanowire since it serves as a waveguide) needs to be much larger, as it is required to support the optical mode into which photons from the radiative recombination of the carriers in the dot emit [16].

Previously such a two-step growth process was applied to embed quantum dots within waveguiding nanowires using temperature to control relative incorporation rates [13]. We found that at sufficiently high growth temperatures (T∼500 °C), incorporation at the metal/semiconductor interface can be entirely suppressed [12] and growth occurs solely in the annulus of exposed substrate. More recently, we have used the Group V flux to manipulate relative incorporation rates [17]. Below we expand on the use of Group V-controlled migration lengths to grow tailored nanowire tapers and how the resulting structures are influenced by the geometry of the SA-VLS nanowire template.

## 3. Results and Discussion

The shape of the grown nanowires can be understood through an appreciation of the relative incorporation rates/diffusion lengths of the group III species (indium in our case) on the different crystal surfaces. The surfaces of importance here are:

(i) The Au/InP interface
(ii) The sidewalls of the nanowire
(iii) The substrate surface surrounding the nanowire

**Figure 3:**
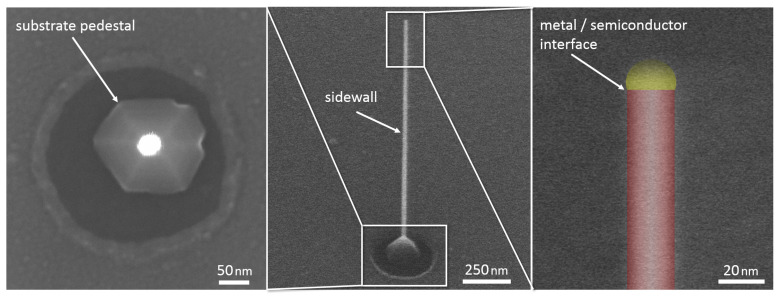
SEM images of a template nanowire showing incorporation sites. Middle and right panels are views at 45° whilst left panel is a plan view. Right panel is in false colour.

**Figure 4:**
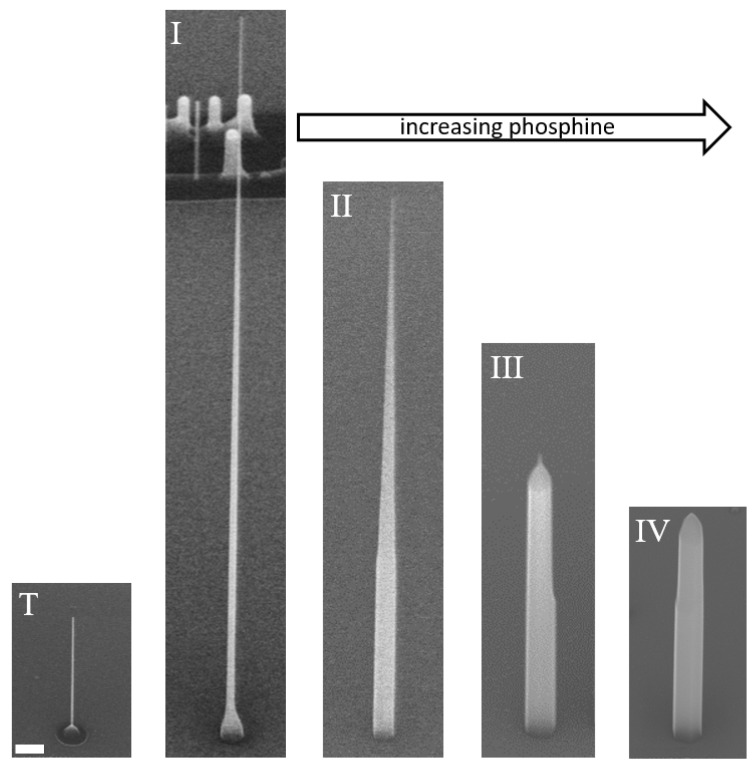
SEM images viewed at 45° of nanowires grown under different cladding conditions. Image T corresponds to the nanowire template on which the cladding material is grown. Images I through IV correspond to nanowires clad using different PH${}_{3}$ flow rates, from 3 to 15 sccm, respectively. Scale bar is 300 nm.

The final geometry of the nanowire is determined by the competition between incorporation on these different surfaces, which can be controlled by the growth conditions used. To grow the nanowire core the conditions are chosen to have a very high incorporation rate at the Au/InP interface and low incorporation rate on the nanowire sidewall (by having a long diffusion length). This means that indium deposited on the sidewall will preferentially diffuse to, and incorporate at, the Au/InP interface or the substrate surface surrounding the nanowire. This results in the structure shown in Figure 3 where an untapered nanowire is shown with a pedestal at the base. When the nanowire becomes significantly longer than the diffusion length of In on the sidewall, typically a few microns, then radial growth and tapering is observed, see image I in Figure 4. If, on the other hand, the incorporation rate at the Au/InP interface can be suppressed relative to that on the sidewall, a radial growth mode can be encouraged. By controlling the relative axial to radial growth rates the geometry of the final nanowire can be engineered.

Here we use a combination of Group V flux and substrate temperature to adjust the relative axial to radial growth rates. Additional versatility is obtained through control of available incorporation sites by modifying the structural parameters of the template i.e., the length $L_{c}$ and diameter $D_{c}$ of the template nanowire and the diameter of the hole in the oxide mask, $D_{h}$ (see Figure 2d). The interdependent roles of Group V flux and nanowire template geometry in determining the photonic nanowire structure are discussed below.

### 3.1. Group V Flux

In this section we study the role of the Group V flux on the nanowire geometry. Four samples were grown (I, II, III, IV) in which the same growth conditions were used for the nanowire template but different PH${}_{3}$ fluxes during growth of the shell. The growth conditions used are shown in Table 1. The templates were prepared using a 12 to 16 s oxide mask wet-etch for hole sizes of $D_{h}∼250$ nm and 5 nm of deposited gold for a catalyst diameter of $D_{c}=20$ nm. The template nanowire growth step was carried out at a temperature of 435 °C using a flow rate, $R_{{\mathrm{PH}}_{3}}=3$ sccm for PH_3_ which are the conditions that promote VLS growth. The growth time was 23 min corresponding to a planar equivalent growth thickness $t_{p}=29$ nm determined from calibration growths on (100) substrates at 500 °C. These growth conditions produce $L_{c}=1.7$ μm long untapered template nanowires such as the one shown in image T of Figure 4.

Sample I was clad using the same growth conditions that were used to grow the nanowire template with an additional planar equivalent growth thickness $t_{p}=46$ nm of InP deposited using a PH_3_ flow rate of $R_{{\mathrm{PH}}_{3}}=3$ sccm. Here we define cladding as any material deposited after the growth of the template nanowire. The resulting nanowire, shown in image I of Figure 4, has a small pedestal at the base and then tapers from 60 nm to 20 nm over a length of 10.7 μm. This geometry is expected when material incorporation occurs primarily at the metal/semiconductor interface and the substrate surface rather than the nanowire sidewall. As mentioned above, growth of the nanowire template is carried out under conditions that promote this VLS growth. As the nanowire becomes longer than the In adatom migration length on the sidewall a slight taper is developed as material incorporates on the sidewalls prior to reaching the metal/semiconductor interface or the substrate. The pedestal at the base will typically grow to fill in the opening in the oxide mask and then increase in height with vertical sidewalls through preferential incorporation at the step edges at the top of the pedestal [18].

Images II through IV of Figure 4 show the effect of the Group V flux used during cladding deposition. For these growths, the cladding temperature was slightly higher, 450° compared to 435°, which does not significantly alter the nanowire geometry [12]. Image II shows the resulting nanowire geometry when the cladding is deposited with a PH_3_ flow rate of $R_{{\mathrm{PH}}_{3}}=9$ sccm. The nanowire is shorter, even though an additional $t_{p}=113$ nm of cladding material was deposited (see Table 1). The top still tapers to 20 nm but over a shorter length. Tapering is a direct consequence of simultaneous radial and axial growth, and the ratio between the two will determine the taper angle, the larger the ratio the larger the angle. This change in relative incorporation rate results from a reduction of the incorporation rate at the metal/semiconductor interface and a reduced In adatom migration length on the sidewall. A taper also results in a high density of step edges which further reduces the diffusion length of indium on the sidewalls. The pedestal at the base is replaced by an untapered base section with diameter $D_{h}$ (e.g., the diameter of the nanowire has filled in the opening in the oxide mask). The diffusion length on the untapered sidewall section ($\left\{11\bar{2}0\right\}$ or $\left\{1\bar{1}00\right\}$ facets) is still long enough for material to reach and incorporate at the tapered section which allows the base diameter to be constrained by the oxide mask.

Increasing the PH_3_ flow rate further (and consequently reducing the axial growth rate) results in even shorter nanowires until the untapered base makes up the entirety of the nanowire, as in images III and IV in Figure 4. At this point growth is dominated by incorporation directly on the $\left\{11\bar{2}0\right\}$ or $\left\{1\bar{1}00\right\}$ facets that form the sidewalls of the nanowire as well as the steps which are now located at the nanowire tip. Under these conditions, the nanowire is no longer constrained by the oxide mask and the diameter can exceed $D_{h}$. We also note that the gold catalyst no longer seems to play any significant role in the growth dynamics, a similar result is observed when using high growth temperatures [12]. The above results are summarised in Table 1.

**Figure 5:**
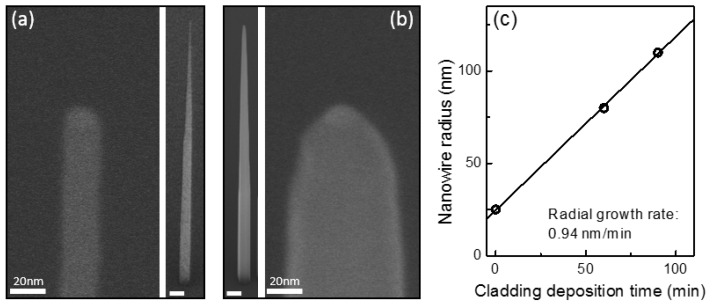
(**a**) SEM image of full structure (right panel) and close-up of tip (left panel) of a nanowire clad using a PH${}_{3}$ flow rate of 9 sccm. (**b**) SEM image of full structure (left panel) and close-up of tip (right panel) of a nanowire with a first cladding as in (**a**) plus a second cladding using a PH${}_{3}$ flow rate of 15 sccm. Scale bars in images of the full structure are 300 nm. (**c**) Nanowire radius as a function of the growth time used for the second cladding.

This PH_3_ flux-meditated control of the relative incorporation rates can be used to fine tune the nanowire geometry using a multi-step cladding process. For example, in Figure 5a we show a nanowire clad as in sample II but using a shorter template nanowire ($t_{p}=19$ nm, $L_{c}=1.1$ μm). The nanowire has a base diameter of 200 nm, a tip diameter 20 nm and a height of 7.7 μm, very similar to that in image II of Figure 4 but slightly shorter. Figure 5b shows the nanowire geometry obtained if an additional $t_{p}=64$ nm is then deposited at a higher PH_3_ flux (e.g., the growth conditions used to clad sample IV). The resulting nanowire has a similar height (7.2 μm) but the diameter has increased almost uniformly along its length, to $D_{b}=250$ nm at the base and $D_{t}=65$ nm at the tip. This demonstrates that a PH_3_ flow rate of 15 sccm produces pure radial growth. By measuring the nanowire diameter as a function of the growth time of the second cladding, we calculate a radial growth rate of ∼1 nm/min, see Figure 5c under these conditions.

### 3.2. Template Geometry

The structure of the photonic nanowire may be further manipulated depending on the detailed geometry of the initial nanowire template. In the following we consider the influence of the template geometry, in particular the template nanowire height, $L_{c}$, and catalyst diameter, $D_{c}$, as well as the size of the opening in the oxide mask, $D_{h}$, in determining the geometry of the photonic nanowire.

We discuss first the influence of the diameter of the oxide opening, $D_{h}$. The size of the opening allows one to control the base diameter of the photonic nanowire. This diameter is crucial in designing photonic nanowires that provide optimal overlap between embedded emitters and guided optical modes in the nanowire [16]. The required diameter, *D*, will depend on the emission wavelength of the emitter, λ, according to D/λ∼0.25 [16]. Although $D_{h}$ can be readily controlled using wet-etch time [19], to obtain a corresponding photonic nanowire diameter *D* over a sufficiently long section of the nanowire to adequately confine the desired optical mode requires optimisation of the growth conditions used in the cladding process.

**Figure 6:**
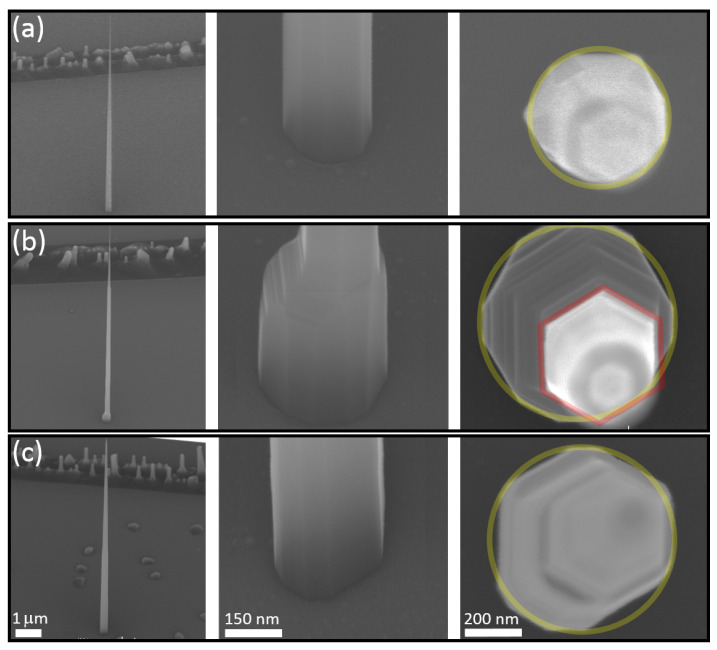
(**a**) SEM images of the full nanowire (left panel), the base (middle panel) and a top view (right panel) for growth on a mask prepared using a 20 s wet-etch. (**b**) Same as (**a**) for a mask prepared using a 24 s wet-etch. (**c**) Same as (**b**) with an additional $t_{p}=64$ nm of cladding deposited. Yellow circles indicate the size of the oxide opening. Red hexagon in (**b**) shows the base of the uniformly tapered section of the nanowire.

As an example, Figure 6a shows a nanowire clad as in sample II but grown on a mask prepared using a 20 s oxide etch. The geometry of the nanowire is very similar to that shown in image II of Figure 4 but with a slightly larger base diameter of 230 nm. Repeating the growth on a mask prepared using a 24 s oxide etch produces the nanowire shown in Figure 6b. Although the base diameter has increased to 325 nm, this only extends a few hundred nanometers above the substrate, after which the diameter abruptly decreases to 195 nm then tapers to 20 nm over the length of the nanowire. As shown in Section 3.1, the cladding process is not simply related to the adatom migration lengths but also requires consideration of preferential incorporation at step edges produced at the base of the nanowire due to substrate growth in the annulus of exposed InP. To obtain a more uniform taper in a nanowire having a large base diameter simply requires additional material deposition at an appropriate PH_3_ flux. This is shown in Figure 6c where an additional $t_{p}=64$ nm of InP was deposited on a structure as in Figure 6b using $R_{{\mathrm{PH}}_{3}}=15$ sccm.

We consider next the role of the height $L_{c}$ and diameter $D_{c}$ of the template nanowire in determining the photonic nanowire geometry. When growing using a low group V flux there is a strong dependence of the axial growth rate on catalyst diameter, $D_{c}$, due to geometric effects on the collection of In in the catalyst [12]. This means that the nanowire height, $L_{c}$, will depend not only on the amount of material deposited but also on $D_{c}$. Specifically, increasing the catalyst diameter produces shorter nanowires. In that using a different value for $D_{c}$ simply results in a nanowire with a different height, the role of the nanowire template diameter on the photonic nanowire geometry is the same as that of $L_{c}$.

In what follows we highlight the effect of *D*${}_{c}$, and hence $L_{c}$, on the nanowire taper when using growth conditions targeting tapered geometries similar to sample II in Figure 4. We use a 12 s oxide mask etch and a $t_{p}=29$ nm template growth step at 420 °C followed by a $t_{p}=113$ nm cladding growth step using $R_{{\mathrm{PH}}_{3}}=9$ sccm. To obtain template nanowires with different diameters, we pattern a linear array of holes in the electron-beam resist (step I in Figure 2b), incrementally increasing the hole size by increasing the electron-beam dose. The increments are adjusted to produces an increase of 2 nm in the catalyst diameter after lift-off (step IV in Figure 2b).

**Figure 7:**
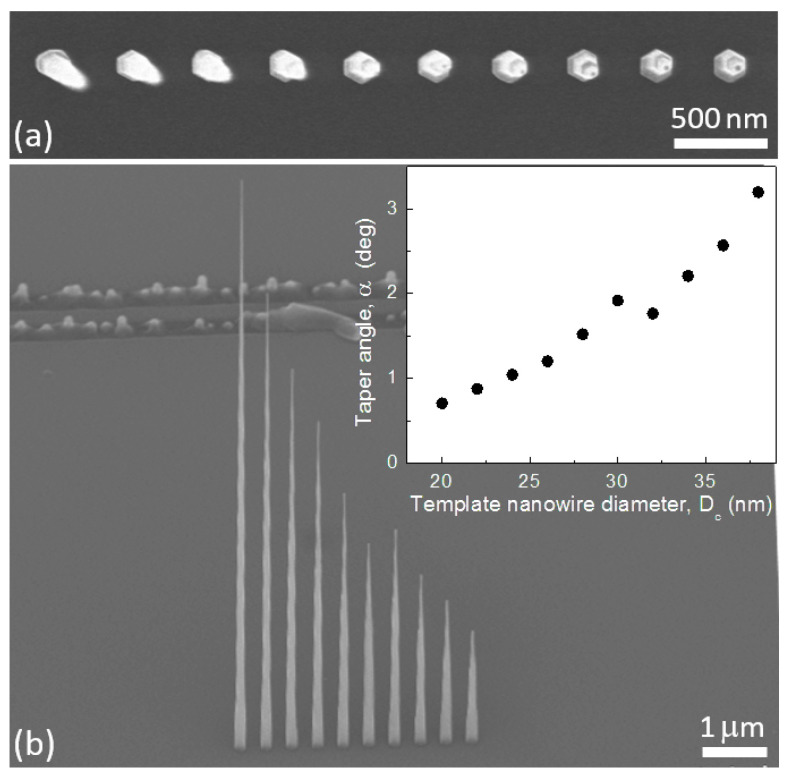
(**a**) Plan- and (**b**) 45°-view SEM images of a linear array of nanowires where the catalyst diameter, $D_{c}$, and hence template nanowire diameter increases from 20 nm (left) to 38 nm (right) in 2 nm increments. Inset in (**b**) shows the dependence of the taper angle α on $D_{c}$.

The resulting linear array of nanowires is shown in Figure 7. The nanowires are uniformly tapered with base diameters $D_{b}$ defined by $D_{h}$ and tip diameters $D_{t}$ defined by the catalyst diameter, which increases from 20 nm (left) to 38 nm (right). The dominant effect of decreasing $L_{c}$ (i.e., increasing $D_{c}$) is a reduction of the nanowire taper $T=\left(D_{b}-D_{t}\right)/L_{T}$ where $L_{T}$ is the taper length. This is quantified in the inset of the figure which shows the calculated taper angle α=atan(T/2) as a function of the template nanowire diameter. This behaviour is a direct consequence of the taper angle dependence on the ratio of axial to radial growth rates. Decreasing the axial growth rate by increasing the Au particle diameter causes an increase in the taper angle.

### 3.3. Optical Characterization

The main incentive for controlling the taper of the photonic nanowire is to reduce the far-field emission angle of the guided optical mode via its gradual expansion. To gain insight into how the emission profile can be manipulated by tailoring the nanowire geometry, we calculate the far-field radiation pattern for different nanowire tapers. Simulations of the HE${}_{11}$ mode propagation along the nanowire taper were performed using finite difference time domain methods and the calculated electric field distribution was transformed into the far-field to analyze the numerical aperture S${}_{NA}=\sin\left(\theta \right)$ of the nanowire source.

**Figure 8:**
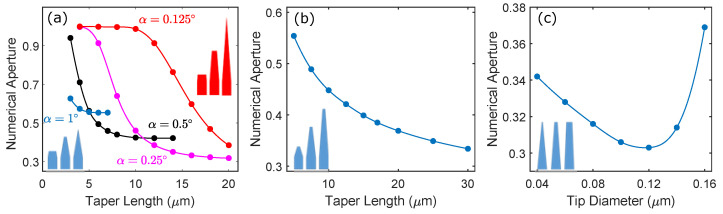
(**a**,**b**) Calculated numerical aperture of the nanowire source S${}_{NA}$ as a function of taper length $L_{T}$ for different taper angles α. In (**a**), the tip diameter $D_{t}$ varies for each value of α whilst in (**b**), $D_{t}$ is fixed and α varies. (**c**) S${}_{NA}$ as a function of $D_{t}$ for a fixed taper length of $L_{T}=20$ μm.

Figure 8 shows the calculated dependence of S${}_{NA}$ on the taper parameters $L_{T}$, $D_{t}$ and α defined in Figure 1a. The simulations were performed with λ=950 nm and $D_{b}=250$ nm. In Figure 8a we show the effect of the taper length $L_{T}$ on the far-field for a fixed taper angle α. In this case, the tip diameter $D_{t}$ also varies, necessarily decreasing as $L_{T}$ increases in order to maintain a fixed α, illustrated schematically by the insets. The figure shows four data sets, each set corresponding to the dependence of S${}_{NA}$ on $L_{T}$ for tapers with a different α. For small values of the taper length, S${}_{NA}$ tends to one, indicating a strongly diverging beam that is unsuitable for coupling to collection optics. As the taper length increase, S${}_{NA}$ is initially insensitive to the changing length, indicating that the HE${}_{11}$ mode is still well confined, but then eventually starts to diminish. The taper length at which S${}_{NA}$ starts to drop increases with decreasing α, since the nanowire diameter at which the HE${}_{11}$ mode is not well confined is reached at longer taper lengths for smaller taper angles. For sufficiently long tapers and small taper angles, arbitrarily low values of S${}_{NA}$ can be achieved.

In Figure 8b we show the variation of S${}_{NA}$ with increasing taper length $L_{T}$ for a fixed tip diameter $D_{t}$ (i.e., truncated tapers). Since fixing the diameter necessarily reduces α as $L_{T}$ is increased, the observed trend is consistent with Figure 8a i.e., long tapers and low taper angles produce low S${}_{NA}$ values. Finally, in Figure 8c, we show the dependence of S${}_{NA}$ on the diameter of the nanowire tip, $D_{t}$, for a fixed taper length of $L_{T}=20$ μm. In this case, a minimum S${}_{NA}∼0.3$ is observed at a tip diameter $D_{t}$ of approximately 0.12 μm, corresponding to a taper angle α of approximately 0.19°. This minimum in the numerical aperture that can be achieved is somewhat surprising at first glance, but the phenomenon is seen clearly in Figure 8a also, where a nanowire of taper length $L_{T}=14$ μm has a smaller S${}_{NA}$ for a 0.25° taper angle than for 0.125° or 0.5°.

To compare with experiment, we use a quantum dot, embedded near the base of the nanowire, to populate the HE${}_{11}$ mode [20]. The dot is incorporated by switching from PH${}_{3}$ to AsH${}_{3}$ for 3 s during the growth of the template nanowire to produce an InAs*_x_*P${}_{1-x}$ section ∼5 nm thick with x∼25%. The photoluminescence from the dot is measured at 4 K in a closed-cycle He cryostat using a grating spectrometer and a liquid nitrogen-cooled CCD. Using the growth conditions above produces dots with exciton photon emission around λ∼950 nm.

**Figure 9:**
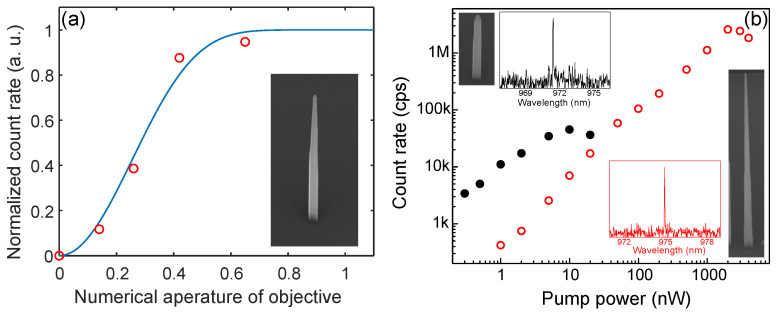
(**a**) Normalized exciton photon emission rate measured at saturation as a function the NA of the collection objective (open circles) for the nanowire device shown in the inset. The blue line is the calculated power, $P_{t}\left(\theta \right)$, transmitted across the base of a cone having an apex angle 2θ using $S_{NA}=0.53$. (**b**) Count rates of single exciton lines as a function of excitation power for a quantum dot embedded in an untapered (solid black circles) and conically tapered (empty red circles) nanowire. Top left (bottom right) insets show an SEM image and a PL spectrum for the untapered (tapered) nanowire.

A rigorous determination of the angular emission distribution requires measurement of the 3D far-field profile, using for example, Fourier microscopy techniques [21]. One can also estimate the divergence of the mode from the dependence of the emitted photon count rate on the NA of the objective used for collection. Figure 9a shows the photoluminescence counts from exciton recombination in the dot collected using different NA objectives. For each objective, the excitation power was adjusted to saturate the count rate of the exciton photons, in this manner guaranteeing that the excitation rates were identical. To estimate the divergence of the emitted beam, we model the system as a Gaussian mode and calculate the collection rate as a function of angle, neglecting any reflection that may occur from the nanowire tip, see Ref. [9]. In Figure 9a, the best fit to the data gives S${}_{NA}=0.53$. For this nanowire geometry ($L_{T}=4.72$ μm, α=0.84, see inset in the figure) the estimated value of S${}_{NA}$ is consistent with the calculations of Figure 8a.

The impact of the nanowire geometry on the collection efficiency is demonstrated in Figure 9b where we compare the count rates from quantum dots embedded in two extreme nanowire geometries. In one device, the dot is incorporated in short nanowire with a truncated taper (top-left inset of the figure) whilst in the second device, the nanowire is optimised for low S${}_{NA}$ with a long, nearly conical taper (bottom-right inset). In particular, the former structure has a taper length of $L_{T}=3.2$ μm and taper angle of α=0.42 for which an $S_{NA}$ approaching 1 is predicted, whilst for the later, $L_{T}=12.8$ μm and α=0.37 for a predicted $S_{NA}∼0.4$. For both devices, the quantum dots have exciton photon emission around λ∼970 nm (see insets) and are incorporated within nanowires having similar base diameters $D_{b}∼250$ nm, hence comparison of the count rate at saturation is a measure of the relative collection efficiency between the two devices. Using an objective with NA=0.42, the count rate from the optimised structure is 57 times brighter compared to the unoptimised device. This indicates that a significant fraction of the emitted beam from the latter device is directed at angles larger than the acceptance angle of the collection objective ($\sin^{-1}\left(\mathrm{NA}\right)=$24.8°), consistent with the high NA of the source.

Finally, we demonstrate an on-demand single photon source based on a single quantum dot embedded in a photonic nanowire having a geometry optimised for high collection efficiency. We use the photonic nanowire structure shown in the bottom-right inset of Figure 9b, which was previously employed to demonstrate 43% collection efficiency [22]. This source efficiency is obtained without the use of a back mirror meaning that 86% of the photons directed towards the taper are collected. To verify single photon operation, a use a Hanbury Brown and Twiss setup. We measure the temporal correlation of emitted exciton photons using pulsed above-band excitation at a repetition rate of 20 MHz. Exciton photons are first filtered and then split into two beams to be measured by two single photon detectors. The arrival time at each detector is register using counting electronics to build a histogram of the delay times between detection events on the first and second detector, $\tau =t_{2}-t_{1}$.

**Figure 10:**
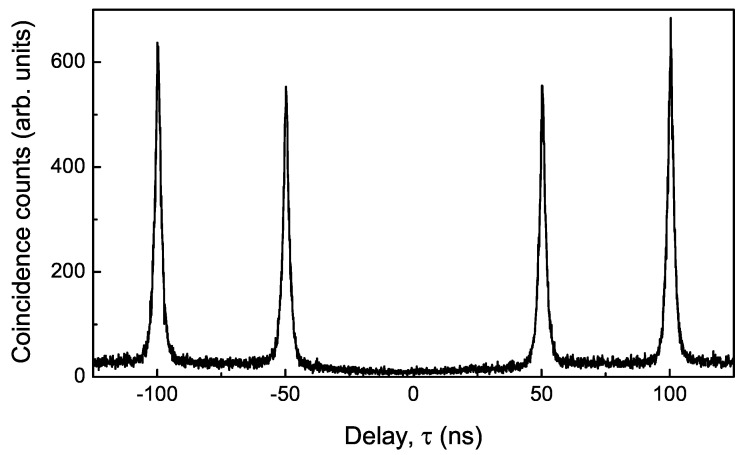
Coincidence counts of exciton photons emitted from a nanowire quantum dot embedded in an optimised photonic nanowire waveguide.

A histogram of the raw coincidence counts is shown in Figure 10 where negligible counts are observed at a delay time τ=0. The complete absence of the zero delay peak is a signature of high purity single photon emission i.e., for each excitation pulse, one and only one exciton photon is emitted. The negligible probability of observing multi-photon emission events is a consequence of using a structure that contains one and only one quantum emitter. This is in contrast to devices employing ensembles of randomly nucleated dots which may suffer spectral pollution from nearby quantum dots, requiring the use of resonant pumping techniques and/or cavity structures [3] to reach similar levels of single photon purity.

## 4. Conclusions

In summary, we have demonstrated the versatility of the SA-VLS epitaxial growth technique for controlling material incorporation sites during growth of III-V nanowire structures. Using appropriate growth conditions, the geometry of nanowire can be tailored to suit the needs of a particular application. We have applied the technique to grow nanowires having geometries capable of emitting highly directional beams that are easily collected with limited numerical aperture optics. As an example, we demonstrate a high purity single photon source based on a quantum dot embedded in a photonic nanowire waveguide where the geometry of the photonic nanowire is optimised for efficient collection using objectives with modest numerical apertures.

## Figures and Tables

**Table 1:** Growth conditions used for cladding and corresponding nanowire geometries for the template nanowire T and samples I through IV in Figure 4.

Sample	PH${}_{3}$ Flow Rate (sccm)	Temperature (°C)	Cladding Thickness $t_{p}$ (nm)	Height (m)	Base Diameter (nm)
T	—	—	0	1.7	20
I	3	435	45	10.7	60
II	9	450	113	7.6	220
III	12	450	149	4.1	305
IV	15	450	149	3.3	300

## Data Availability

The data that support the findings of this study are available from the corresponding author upon request.
